# Supplementary material for: Rewiring Mood: Precision Psychobiotics as Adjunct or Stand-Alone Therapy in Depression Using Insights from 19 Randomized Controlled Trials in Adults
Source: Nutrients. 2025 Jun 17;17(12):2022. doi: 10.3390/nu17122022 (PMC12196188; doi:10.3390/nu17122022)
Supplement: Supplementary file 1 [file nutrients-17-02022-s001.zip › nutrients-3677688-supplementary.pdf]

Table S1: Psychometric tools used in each study

Score	Items	Scoring per item	Target population	Purpose	Cut-off points
BDI-II	21	0-3	Adults and adolescents (>13 years)	presence and severity of depressive symptoms	>14
HADRS	14	0-3		depression and anxiety	>8
HAM-D	17 (or 21/24)	0-4		somatic and cognitive symptoms	>14
DASS-21	21	0-3	Adults and adolescents (> 14)		>5 (or doubled >10)
BAI	21	0-3	Adults and adolescents	Somatic symptoms of anxiety	>16
SCL-90	90	0-4	Adults	psychological distress and symptom patterns across multiple domains	No cut off points
Z-SDS	20	1-4	Adults	presence and severity of depression	50
HDRS-24	24	0-4	Adults	Suspected/confirmed depression	>19
MADRS	10	0-6		severity of depressive symptoms and response to treatment	>20
BPRS	18 or 24	1-7		depression, anxiety, hallucinations, and thought disorder	No universal cut-off scores
GSRS	15	1-7		Used primarily for symptom monitoring and treatment response evaluation in GI disorders.	No cut-off
PHQ-9	9	0-3	Adults		>10
STAI	40	1-4	Adults and adolescents		>20
IDS	30	0-3	Adults		>14
HAMA	14	0-4	Adults		>18
GAD-7	7	0-3	Adults and adolescents		>10
CESD-R	20	0-3	Adults		>16
SCID-5-RV				diagnostic classification of mental disorders	
Z-SAS	20	1-4	Adults	Severity of anxiety symptoms	>45
